# Development of a Chitosan/PVA/TiO_2_ Nanocomposite for Application as a Solid Polymeric Electrolyte in Fuel Cells

**DOI:** 10.3390/polym12081691

**Published:** 2020-07-29

**Authors:** Elio Enrique Ruiz Gómez, José Herminsul Mina Hernández, Jesús Evelio Diosa Astaiza

**Affiliations:** 1 Grupo de Investigación en Estudios Aeroespaciales - GIEA, Escuela Militar de Aviación Marco Fidel Suárez, Carrera 8 No. 58-67, Cali 760004, Colombia; elio.ruiz@emavi.edu.co; 2 Grupo Materiales Compuestos - GMC, Universidad del Valle, Calle 13 No 100-00, Cali 76001, Colombia; 3 Departamento de Física, Universidad del Valle, Cali A.A 25360, Colombia; jesus.diosa@correounivalle.edu.co; 4 Centro de Excelencia en Nuevos Materiales (CENM), Universidad del Valle, Cali A.A 25360, Colombia

**Keywords:** solid polymer electrolyte, titanium oxide, chitosan, polyvinyl alcohol, fuel cell

## Abstract

The influence of the incorporation of nanoparticles of titanium oxide (TiO_2_) at a concentration between 1000 and 50,000 ppm on the physicochemical and mechanical properties of a polymer matrix formed from a binary mixture of chitosan (CS) and polyvinyl alcohol (PVA) at a ratio of 80:20 and the possibility of its use as a solid polymeric electrolyte were evaluated. With the mixture of the precursors, a membrane was formed with the solvent evaporation technique (casting). It was found that the incorporation of the nanoparticles affected the moisture absorption of the material; the samples with the highest concentrations displayed predominantly hydrophobic behavior, while the samples with the lowest content displayed absorption values of 90%. Additionally, thermogravimetric analysis (TGA) showed relatively low dehydration in the materials that contained low concentrations of filler; moreover, differential scanning calorimetry (DSC) showed that the nanoparticles did not significantly affect the thermal transitions (Tg and Tm) of the compound. The ionic conductivity of the compound with a relatively low concentration of 1000 ppm TiO_2_ nanoparticles was determined by complex impedance spectroscopy. The membranes doped with a 4 M KOH solution demonstrated an increase in conductivity of two orders of magnitude, reaching values of 10^−6^ S·cm^−1^ at room temperature in previously dried samples, compared to that of the undoped samples, while their activation energy was reduced by 50% with respect to that of the undoped samples. The voltage–current test in a proton exchange membrane fuel cell (PEMFC) indicated an energy efficiency of 17% and an open circuit voltage of 1.0 V for the undoped compound, and these results were comparable to those obtained for the commercial membrane product Nafion^®^ 117 in evaluations performed under conditions of 90% moisture saturation. However, the tests indicated a low current density in the undoped compound.

## 1. Introduction

The energy resources derived from oil produce environmental pollution; thus, currently, the use of alternative environmentally friendly energy-generating technologies is being implemented. Fuel cells are electrochemical devices that generate efficient electrical energy. These devices have the ability to continuously convert the chemical energy of a fuel into electrical energy. Polymer electrolyte membranes (PEMs) are a fundamental part of proton exchange membrane fuel cells (PEMFCs) and direct methanol alkaline fuel cells (DMAFCs). The authors Tripathi and Shahi [1] highlight three main aspects in the development of PEM: the good mechanical behavior of the membrane, the effect of the ceramic filler on the polymer matrix in balancing its hydrophilic nature, and the chemical activation (doping) of the membranes to produce PEMs with high efficiencies and low costs. In this regard, Wang and Wang [2] argue that the water retention of membranes at high temperatures is favored with adequate dispersion of inorganic filler. One of the aspects mentioned by Ayse and Bozkurt [3] for achieving optimal performance from a PEM is the adequate hydration of the ionomeric structure of the polymer. The authors mention the study of chitosan (CS) as a low-cost and environmentally friendly biopolymer with wide potential as an electrolyte. Additionally, polyvinyl alcohol (PVA) is a biodegradable synthetic polymer that presented high ionic conductivities when the polymeric matrix was doped with hypophosphorous acid in studies by González and Vargas [4]. Gonçalves et al. [5], Ali and Gherissi [6], Aziz et al. [7], Benítez et al. [8], and Quintana et al. [9] reported that blending of chitosan with synthetic polymers, such as PVA, is a convenient method for preparation of synthetic biodegradable polymers having versatile properties such as good water absorbance and enhance mechanical properties. Additionally, Gonçalves et al. [5] found important information on the role of CS on the mechanical properties of the blended films in the composition between 70:30 to 85:15. Moreover, it is reported that the excess of PVA limits it use due to its high solubility in water. As a consequence, in this work we have selected for our blended films the PVA:CS ratio of 80:20.

In the last decade, numerous research efforts have been made in the development of new materials with the solid polymeric electrolyte (SPE) nature described by Habiba et al. [10]. In the present work, TiO_2_ nanoparticles were dispersed in the compound, doped and undoped with potassium hydroxide (KOH) for the purpose of producing a solid plasticizing effect and improving the physical and chemical properties of the membranes produced by the casting technique. Additionally, a voltage–current test performed in a PEMFC was correlated with the thermogravimetric characteristics of the binary matrix. In future work, the effect of chemical crosslinking on the stability of the material in aqueous solutions will be performed.

## 2. Materials

The polyvinyl alcohol (PVA) used displayed molecular weight Mw values between 85,000–124,000 g/mol and a hydrolysis of 99%. The chitosan used displayed Mw values of 50,000–124,000 g/mol and a degree of deacetylation between 75–85%. The titanium dioxide (TiO_2_) nanoparticles were of the anatase form with an average particle size of 21 nm. The acetic acid used had a purity of 99.9%, while that of the potassium hydroxide (KOH) used was 90%. All reagents used in the investigation were purchased from Sigma Aldrich and were used as received without further purification.

## 3. Experimental Procedure

### 3.1. Preparation of PVA/CS-TiO_2_ Polymeric Membranes

**Figure 1:**
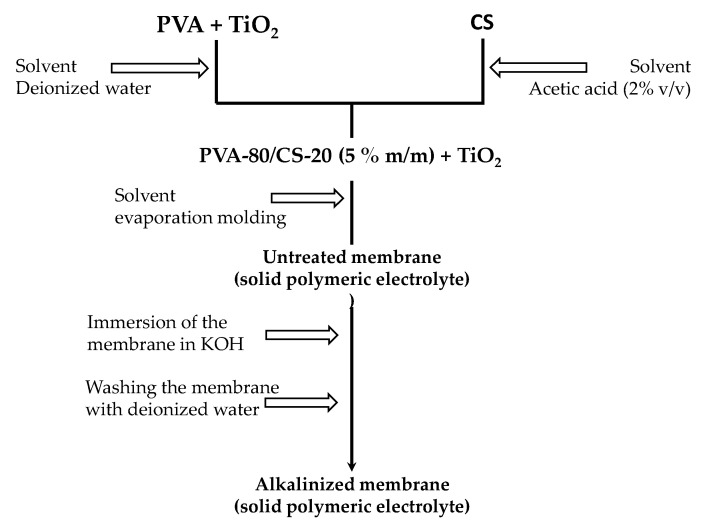
Diagram of the preparation of the alkaline membrane. Abbreviations: PVA, polyvinyl alcohol. CS, chitosan.

To prepare PVA/CS-TiO_2_ polymeric membranes with a PVA/CS 80/20 ratio (by mass) and TiO_2_ nanoparticles at a concentration ranging between 1000 and 50,000 ppm of the total solute, a solution was prepared with a mass concentration of 5%. The process began with the dispersion of the lowest concentration of the ceramic filler (5.0 mg of TiO_2_) in a glass with distilled and deionized water using Elma E30H ultrasound equipment for 30 min. Subsequently, 3996 g of PVA was added under magnetic stirring for 8 h at 50 °C, followed with a heating ramp of 15 °C/hour until a maximum temperature of 110 °C was reached to obtain a homogeneous viscous solution. Additionally, a 2% by volume solution of acetic acid in water was prepared, and then 3% CS (by mass) was incorporated, maintaining magnetic stirring at room temperature for 24 h. After this, the solutions of PVA + TiO_2_ and CS were mixed under magnetic stirring for 30 min at room temperature, obtaining a solution with a concentration of 5% by mass. Finally, thin membranes with an average thickness of 0.5 mm were formed through casting using the same method reported in similar studies by Paipitak et al. [11] and Benitez et al. [8] of depositing the solution on a Petri dish and subjecting it to an oven drying process at 37 °C for approximately 48 h. Thereby, the compound named PVA/CS/1000 ppm TiO_2_ was obtained. Following an analogous process, the other membranes with higher concentrations of the filler within the dispersion were obtained: 5000, 10,000, and 50,000 ppm. Then, to carry out the doping process, the membranes were immersed in 2 and 4 M KOH solutions for 24 h. Next, the material was removed from the alkaline solution, and its surface was rinsed with distilled water. Finally, the membranes were dried in an oven at 37 °C for a period of 48 h. Figure 1 schematically shows the procedure followed to obtain the alkalized membranes.

### 3.2. Morphological Characterization

To evaluate the cross-section of the membranes and study their morphology, a JEOL model JSM 6490 LV scanning electron microscope (Tokyo, Japan) was used. Previously, the samples were introduced to liquid nitrogen until they reached a vitreous state, and then the samples were fractured by the application of a shear stress. Subsequently, the surface of the specimens was covered with a gold film by means of a Denton Vacuum Model Desk IV to generate a conductive area. The images were obtained in the backscattered electron mode using an acceleration voltage of 20 kV.

### 3.3. Thermal Characterization (DSC and TGA)

A TA model Q100 differential scanning calorimeter DSC (New Castle, DE, USA) was used to determine the thermal transitions of the membranes (Tm and Tg). Samples with a mass of approximately 5 mg were subjected to a heating rate of 10 °C/min and a temperature range of −80 to 400 °C. Previously, the samples were dehydrated by heating from 30 to 150 °C. The degree of crystallinity of the membranes was estimated using the model presented in Equation (1).

(1) $$X_{C}\left(\%\right)=\frac{∆H_{f}}{\left(w_{PVA}\right)∆H_{f}^{o}}*100$$

where $∆H_{f}^{o}$ is the enthalpy of fusion of 100% crystalline PVA, with a theoretical value of 138.6 J/g as reported by Guan et al. [12]; *∆H_f_* is the enthalpy of fusion of the sample; and $w_{PVA}$ is the mass fraction of PVA used in the mixture. The TGA measurements were performed in a thermogravimetric analysis model 2050 TA using a heating rate of 10 °C/min, a sample mass of approximately 5 mg, and a protective atmosphere with a constant flow of N_2_ gas.

### 3.4. Moisture Absorption

The previously prepared polymeric membranes were placed for 12 h in a balanced chamber at a relative humidity (RH) of 7%. The mass of the dehydrated membranes was recorded as $m_{1}$. Subsequently, the samples were introduced to another chamber with an RH of 90% at a temperature of 25 °C for approximately 100 h. The mass of the hydrated membranes as a function of time was designated $m_{2}$. The percentage of moisture absorption (% H) of the samples was determined by gravimetry using the model presented in Equation (2).

(2) $$H=\left(\frac{m_{2}-m_{1}}{m_{1}}\right)\times 100$$

### 3.5. Complex Impedance Spectroscopy

The study of the electrical conductivity of the samples was carried out using a Wayne Kerr model 6420 impedance meter, and the sample was placed between two gold electrodes. The complex impedance data were taken in the impedance cell in the frequency range of 20 to 5 MHz and a temperature range of 27 to 160 °C with a heating ramp every 10 °C. The response to the applied potential was the electrical resistance (R) of the sample. These values were obtained directly from the impedance graph from the intersection of the semicircle with the real axis of the complex plane. The values of the DC electrical conductivity (σ) of the membranes are obtained using Equation (3).

(3) $$\sigma =\frac{L}{RA}$$

where L is the thickness of the sample and A is the effective cross-section of the sample. The dependence of the electrical conductivity on temperature was analyzed using the Arrhenius model indicated in Equation (4).

(4) $$\sigma =\sigma_{o}exp\left[-\frac{E_{A}}{K_{B}\left(T\right)}\right]$$

where $\sigma_{o}$ is the pre-exponential factor, which is related to the number of charge carriers; *E_A_* is the activation energy, which represents the minimum energy required for an ion to jump from one position to another; and *K_B_* is the Boltzmann constant. Based on the least squares fit, the linearization of Equation (4) is carried out with Equation (5).

(5) $$ln\sigma =ln\sigma_{o}-\frac{E_{A}}{K_{B}\left(T\right)}$$

### 3.6. Mechanical Characterization (Tensile Tests)

The tensile tests were performed on a Tinius Olsen Model H50KS Universal Testing Machine (Uttar Pradesh, India) with a load cell of 1000 N. The samples were cut into rectangular section tapes with a length, width and thickness of 50, 15, and 0.50 mm, respectively, following ASTM standard D882-09 [13]. The strips were fixed to a wedge-type clamp. The stress tests in all cases were performed at room temperature (T = 25 °C) with a displacement speed of 20 mm/min. Prior to the stress tests, the samples were equilibrated at a relative humidity of 50% according to ASTM standard E104-02 [14]. To evaluate each treatment, three samples were used, and the average and standard deviation were reported. The energy at rupture of the membrane (tenacity), which is expressed as energy per volume, was obtained from the area under the stress–strain curve of each sample.

### 3.7. X-ray Diffraction (XRD)

An X’Pert-XRD PANalytical diffractometer (Malvern, UK) was used to collect the diffraction spectra of the samples. Kα Cu radiation (λ = 1.548 Å, 45 kV and 40 mA) was used, with a nickel filter employed to avoid the Kβ line of Cu, and the diffraction intensity at 2θ ranging from 10 to 90° degrees with a step of 0.02° was measured at room temperature.

### 3.8. Current–Voltage Tests

The fuel cell design was based on the cell described in the Fuel Cell Technology Handbook, Hoogers, G., et al. [15]. For this design, graphite diffusers with a coil type channel arrangement and electrodes (0.5 mg/cm^2^) with 60% platinum on Vulcan carbon cloth, reference W1S1009 purchased from the company FuelCellStore (College Station, TX, USA), were used.

For the current–voltage tests of the polymeric membrane in the proton exchange membrane fuel cell (PEMFC), hydrogen gas grade 5.0, and oxygen gas grade 2.8 were used. An approximate flow of 1.6 cm^3^/min of hydrogen was monitored by flowmeters. The efficiency of the fuel cell ($\eta_{e}$) was expressed in terms of the ratio of the average voltage ($V_{mean}$) of the operating cell and the ideal cell voltage ($V_{ideal}$) and ideal fuel cell efficiency ($\eta_{i}$). Since the cell voltage was smaller than the ideal voltage ($V_{ideal}$) due to the losses associated with polarization and ohmic losses, the efficiency of the cell was estimated using the model shown in Equation (6) reported by EG&G TechnicalServices [16].

(6) $$\eta_{e}=\frac{\left(V_{mean}\right)\eta_{i}}{V_{ideal}}$$

The efficiency of the samples is due to ohmic losses and contact losses. In a commercial fuel cell MEA (Electrode Membrane Assembly) it is completely sealed. In our work, we must bear in mind that the fuel cell that was designed must have the power to disassemble, therefore, the MEA is not sealed, which leads to lower efficiencies than the studied PEM.

## 4. Results and Discussion

### 4.1. SEM Morphology

**Figure 2:**
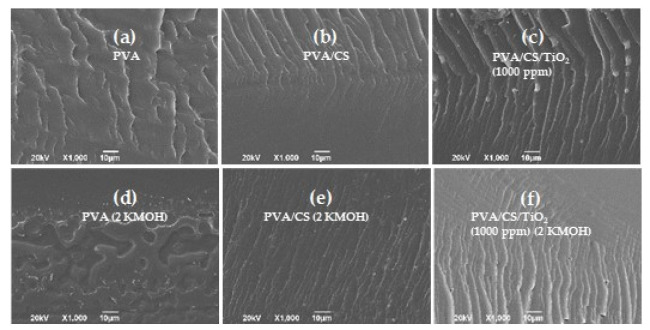
SEM micrographs of membrane cross-sections: (**a**) PVA; (**b**) PVA/CS; (**c**) PVA/CS compound (1000 ppm TiO_2_); (**d**–**f**) the 2 M KOH doped membranes, respectively.

Figure 2 shows the SEM images corresponding to the morphology of the cross-sections of the polymeric membranes. In all cases, a homogeneous surface free of defects is observed. The roughness that is revealed in the images is mainly due to the cross-section preparation process. Similar results were obtained by Cruz et al. [17], who evaluated PVA/CS mixtures with organic and inorganic fillers and found a homogeneously dispersed phase that improved the mechanical, thermal, and conduction properties of the materials.

### 4.2. Moisture Adsorption

**Figure 3:**
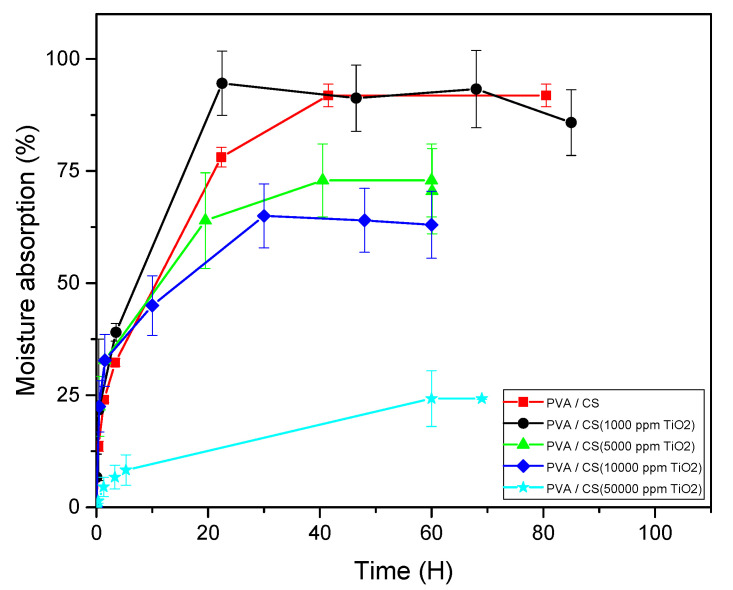
Moisture absorption isotherms for membranes with different TiO_2_ contents.

The moisture absorption curves of the membranes at room temperature are shown in Figure 3. In the case of the PVA/CS binary mixture, an increase of 40% of its mass is observed after five hours of conditioning of the membrane sample in the humidity chamber. Subsequently, in approximately 40 h, an absorption of 90% is reached. This increase in mass is associated with the hydrophilic nature of the polymers used, which have water-soluble polar groups such as the hydroxyl -OH and the amino group -NH_2_. In addition, water is oriented within the polymer matrix by the hydrogen bonds or dipoles generated by interactions with the -OH groups of the polymer and absorbed in the capillary pores as free water. Additionally, an adequate dispersion of the ceramic filler particles can cause some defects or vacancies in the matrix, thus generating a free volume at the interface between these ceramic particles and the polymer chain where the absorption capacity and retention of water molecules of the polymeric compound are improved. When the concentration of TiO_2_ is increased from 1000 to 50,000 ppm, it is generally observed that there is a significant decrease in the absorption of moisture in the polymeric compound. In this regard, Yang et al. [18] showed that polymer membranes (produced by casting) with high concentrations of TiO_2_ or SiO_2_ fillers demonstrate a decrease in water absorption, and they attribute this phenomenon to the nature of these fillers. When incorporating the nanoparticles in the polymeric membrane at the lowest concentration, a behavior similar to that of the unfilled membrane is obtained due to the minimal competition between the dispersed particles and the water in the porous spaces. However, with higher concentrations of TiO_2_, the compound shows significantly lower moisture absorption, reaching an equilibrium absorption value of 20% for the highest concentration of the filler. This behavior is possibly due to the dilution effect of the polar groups of the polymers and to the interference of the ceramic particles in the porous spaces that are commonly occupied by water, resulting in a smaller volume of effective water absorption in the samples studied. It can be argued that because membranes with a TiO_2_ concentration of 1000 ppm do not inhibit the moisture absorption of the compound, its behavior is interesting in the context of its application as a solid polymeric electrolyte.

### 4.3. Thermogravimetric Analysis (TGA)

**Figure 4:**
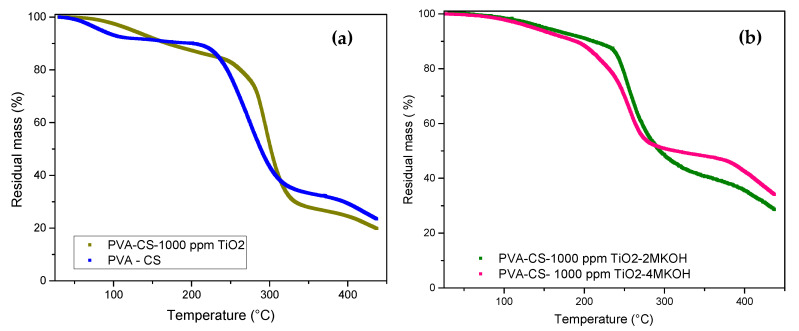
TGA curves for membranes: (**a**) composites. (**b**) KOH-doped compounds.

For a concentration of 1000 ppm of filler in the binary mixture, moisture retention is observed in the compound with respect to the binary membrane formed with the precursors (see Figure 4a). That is, a synergistic effect is evidenced between the binary mixture and the ceramic filler in the thermal properties of the compound. Mollá and Campañ [19] mention that effective dispersions of particles of hygroscopic metal oxides—such as SiO_2_, TiO_2_, and ZrO_2_—in acidic membranes improve both water retention and thermal stability. This phenomenon can be explained by the interaction of the thermally stable ammonium and viable hydroxyl groups in the membranes with the studied filler, producing greater thermal stability in the compound.

Vargas et al. [20] indicated that the TGA results for PVA samples showed relatively high water retention (approximately 5% or more). Figure 4b shows the TGA results for the 2 and 4 M KOH doped compounds, which indicate a decrease in thermal stability compared to those of the undoped compounds. This behavior is possibly due to the interaction of the basic solution with the intra- and intermolecular bonds of the hydrogen bonds of the hydroxyl groups of the precursor polymers in the binary membrane.

### 4.4. Differential Scanning Calorimetry (DSC)

**Figure 5:**
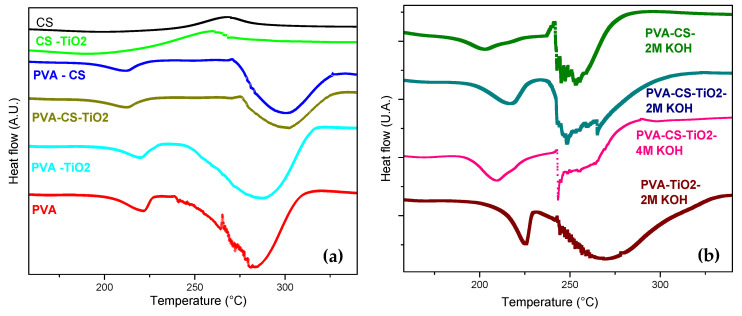
DSC curves for the compound and its precursors: (**a**) undoped; (**b**) doped with KOH.

The thermal transitions for the PVA/CS compound (1000 ppm TiO_2_) and its precursors are estimated by differential scanning calorimetry (DSC), as shown in the thermograms of Figure 5.

As shown in Figure 5a, an endothermic peak is observed for the PVA membrane at a temperature of 203 °C with an enthalpy of 45 J/g, which is attributed to the melting process of the crystalline region. This is in good agreement with the report of González and Vargas [4], who recorded a melting temperature at around 200 °C in a study on the thermal and electrical behavior of a PVA matrix. Likewise, Yang and Chiu [21] indicated a similar temperature of 229 °C for this parameter. At a higher temperature of 263 °C, a second endothermic peak is observed due to polymer decomposition, with an enthalpy of 526 J/g. In the CS thermogram, an exothermic peak is detected at approximately 270 °C with an enthalpy of 90 J/g, which is attributable to the decomposition of the Amina units. These values are close to those reported by Benitez et al. [8]. For the binary PVA/CS mixture, an endothermic peak is observed at a temperature of 194 °C with an enthalpy of 32 J/g, which is attributed to the melting process of the crystalline region. These values are lower than those presented in the PVA, indicating a higher proportion of amorphous structure in the binary membrane. From this behavior, we can infer that the mixture of PVA and CS polymers forms a new polymeric structure. Likewise, Yang and Wang [22] reported that polymers with high crystallinity can have high temperatures and heats of fusion. The author found that the melting point of PVA is higher than that presented in the membranes of the PVA/CS mixture, with melting temperature values of 229 and 224 °C, respectively. This is because the crystallinity of the binary membrane is lower than that of the pure PVA matrix. The degree of crystallinity is calculated using the model described in Equation (1). The crystallinity (χ_C_) estimated for the pure PVA sample is higher than that estimated for PVA/CS, with values of 32% and 29%, respectively. The small difference observed in between the intensities of the endothermic peaks of the binary membrane and the PVA/CS compound (1000 ppm TiO_2_) in the DSC results supports the similarity between the degrees of crystallinity of these samples. Therefore, it is concluded that the degree of crystallinity of the binary membrane is not very sensitive to the content of 1000 ppm TiO_2_. This result is corroborated in the following X-ray diffraction section, where the diffraction spectrum of the PVA/CS mixture slightly changes when the ceramic filling is incorporated. In this regard, González and Vargas [4], in a study on the morphological behavior of the polyelectrolyte formed with the combination of PVA and H_3_PO_2_, reported a very weak effect of TiO_2_ on the melting point of the crystalline part of the material with the nanoparticle contents. Figure 5b shows the DSC thermogram of the compounds doped by immersion in basic solutions of 2 and 4 M KOH. KOH has a noticeable effect on the thermal behavior of the system through the almost consecutive formation of the endothermic peaks of fusion and decomposition. These results support the results of the TGA curves, where lower thermal stability (lower decomposition temperature) is indicated for the compounds doped with the basic solution of 2 and 4 M KOH.

### 4.5. X-ray Diffraction

**Figure 6:**
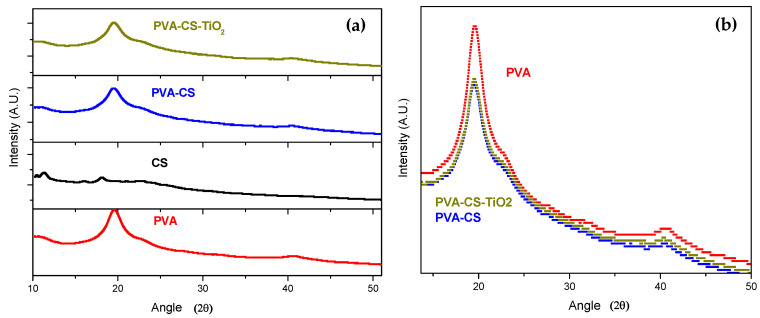
(**a**) X-ray diffraction spectra of precursors and the PVA/CS system (1000 ppm TiO_2_). (**b**) Qualitative comparison of the diffraction peaks.

Figure 6a shows the X-ray diffraction spectra of the compound and its pure precursors. The peak of greatest intensity observed in the pure PVA membrane is located at a 2θ of approximately 20°, indicating a degree of membrane crystallinity with an atactic (random) configuration of the OH groups of the polymer. This diffraction behavior persists in the membrane of the binary mixture. However, as shown in Figure 6b, the intensity of this peak decreases for the binary mixture. This can be interpreted with the criterion proposed by Hodge et al. [23], in which a correlation is established between the peak height and the degree of crystallinity of the polymer; in this case, the degree of amorphousness increases when the CS is incorporated in the PVA matrix. These results are in agreement with those reported by García et al. [24]. The authors state that this phenomenon is due to the decrease in intermolecular interactions between the chains of the mixture of the precursor polymers; thereby, the degree of crystallinity decreases. Notably, in the diffractogram of the compound, which indicates that the incorporation of the filler into the binary matrix does not significantly alter its crystallinity. This is consistent with the behavior of the fusion enthalpy change of the system described in the previous section.

### 4.6. Tensile Tests

**Figure 7:**
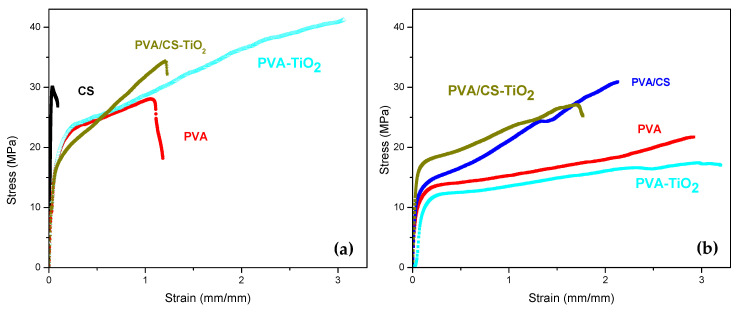
Stress–strain curves: (**a**) PVA/CS compound (1000 ppm TiO_2_) and precursors. (**b**) 2 M-doped KOH membranes. Crosshead speed 20 mm/min.

Figure 7a shows the mechanical behavior of the compound and the membranes produced with the precursors. The value of the Young’s modulus of the compound is approximately 251 MPa, between the 208 and 962 MPa obtained for its precursors PVA and CS, respectively. The PVA sample has an average toughness of 38 MJ/m^3^, which is of the same order as that reported by Guan et al. [12]. Additionally, the CS presents a toughness of 4 MJ/m^3^. The compound exhibits a toughness of 24 MJ/m^3^. The stress–strain curves in Figure 7a,b shows that the maximum strain tends to be greater for the membranes that included TiO_2_ in their formulation, this suggests that the dispersed particles act as solid plasticizer in the material, similar to what happened with the incorporation of the PVA in the CS matrix. The effect of doping with 2 M KOH on the compound and the membranes produced with the precursors is presented in Figure 7b. In the PVA membrane, the toughness increases with respect to the membrane formed with the undoped pure polymer. This may be due to KOH affecting the dipole bonds of the OH groups between the polymer chains. On the other hand, in the compound, the toughness increases by 54% compared to that of the undoped compound. In all the cases studied, the stress at rupture tends to be lower in the KOH-doped membranes. Additionally, the percent elongation of the doped membranes is higher. Mohamad et al. [25] mention that doping with KOH alters the crystalline nature of alkaline solid polymer electrolytes based on PVA and favors the amorphous phase. Likewise, Wan et al. [26] found that the crystalline properties of a chitosan matrix membrane are modified after being doped with KOH due to the breakage of the hydrogen bonds in their polymer chains. These previous results reported by researchers show that the modification of the crystalline phase of polymers affects their mechanical properties.

### 4.7. Complex Impedance Spectroscopy

**Figure 8:**
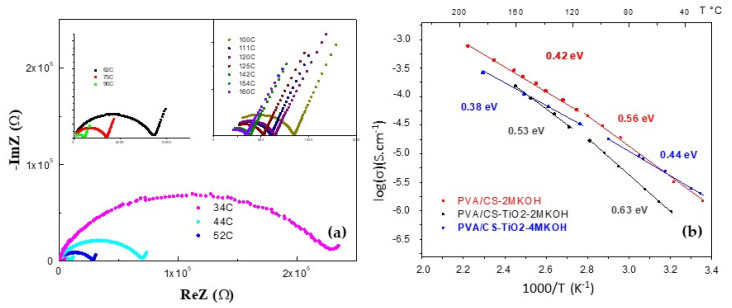
(**a**) Nyquist diagrams, −Z′′ vs. Z′, of the compound PVA/CS (1000 ppm TiO_2_). (**b**) Arrhenius model, compound doped with KOH.

The DC conductivity (σ) is obtained from the electrical resistance (R) and the dimensions of the sample according to the model proposed in Equation (3). The conductivity of the CS membrane at room temperature is approximately 10^−7^ S·cm^−1^, which is within the range reported in the literature. Garcia et al. [24] reported a conductivity of 10^−5^ S·cm^−1^ in a CS membrane. González and Betzabe [27] defined the conductivity of pure CS as ionic due to the presence of free hydroxyl groups and reported values between 10^−9^ and 10^−11^ S·cm^−1^. In addition, the authors reported that the absorption of moisture from the environment can increase the conductivity to 10^−4^ S·cm^−1^. Similarly, Wan et al. [20] stated that chitosan membranes have been used for the active transport of anions in aqueous solutions, achieving conductivities of 10^−2^ S·cm^−1^ with KOH-activated membranes. Additionally, PVA and PVA/CS-based solid electrolyte composite membranes have been reported with conductivities of the order of 10^−2^ S·cm^−1^ González and Vargas et al. [4], Benítez et al. [8], Permana et al. [28], and Quintana et al. [9]. Due to its electrolyte characteristics, CS can also function as a membrane for alkaline fuel cells where hydroxyl transport ions are particularly required. Figure 8a shows the impedance spectra of the compound for various isotherms from 34 to 160 °C. The ionic conductivity of the compound at room temperature is determined to be 10^−8^ S·cm^−1^, which is attributed to the measurements being taken in samples with low humidity. The conduction values found for the membranes at low temperatures are due to the contribution of the liquid phase present in the membrane and to the amorphous phase. At higher temperatures, an increase in conductivity is observed (evidenced in the spectrum by the decrease in R), possibly due to the increase in the amorphous region as the melting point of the compound is approached (with a melting temperature of 190 °C, shown in the DSC analysis). For the doped compound, a conductivity value on the order of 10^−6^ S·cm^−1^ is recorded at room temperature, showing an increase of two orders of magnitude with respect to that of the undoped compound under low humidity conditions.

Figure 8b shows the curves of lnσ as a function of the inverse of the temperature for the membranes doped with KOH. Two regions of thermal activation are observed. The first region corresponds to low temperatures (30–90 °C) and is influenced by the remaining moisture in the sample. In the second region, at higher temperatures (90–160 °C), the ionic jump mechanism dominates, influenced by the flexibility of the polymer chains due to the proximity to the melting temperature of the polymers. The activation energy, Ea, is calculated using the slopes and parameters of the linear fit in each of the aforementioned regions. For the compound, activation energies of 0.96 and 0.33 eV were obtained at low and high temperatures, respectively (not shown in Figure 8b). This is due to the incorporation of PVA since the dispersion of TiO_2_ in the CS matrix produces a redistribution of the free volume in the compound. In addition, a general decrease in activation energy is observed in the samples doped with the 2 and 4 M KOH solutions. This is due to the increase in charge carriers in the matrix of the compound. Note that the samples doped with the 4 M KOH solution show better behavior in terms of diffusion of charge carriers, which is evidenced by their lower activation energies of 0.44 and 0.38 eV at low and high temperatures, respectively.

### 4.8. Current–Voltage Analysis

**Figure 9:**
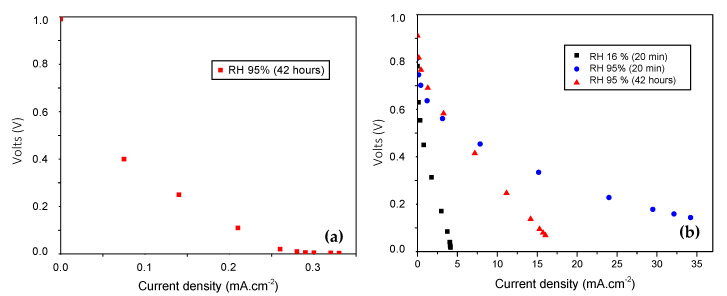
Voltage vs. current density curve for (**a**) undoped compound; (**b**) Nafion^®^ 117.

Figure 9 shows the voltage vs. current density graph for the proton exchange membrane fuel cell (PEMFC) using the solid electrolyte PVA/CS compound (1000 ppm TiO_2_) and Nafion^®^ 117, with different degrees of humidity used in the conditioning of the samples. The membranes are subjected to the following humidification environments: 16% and 95% RH (for a period of 20 min) and 95% RH (for 42 h) according to the Nafion^®^ 117 study protocol and the saturation curves. The calculation of the energy efficiency is performed through the application of Equation (6). The data are acquired under the same operating conditions for all membranes. Figure 9a shows the behavior of the proton exchange membrane fuel cell (PEMFC) with the matrix of the electrolytic compound PVA/CS (1000 ppm TiO_2_). The material only shows electrical stability for the treatment of the highest degree of moisture saturation, achieving an energy efficiency of 16.7% and an open circuit voltage Vca = 967 mV. These results suggest that the matrix is promising as a polymeric electrolytic membrane (PEM). The intrinsic charge carriers of the compound that contribute to ionic conductivity are mainly those provided by chitosan, the conductivity of which is 10^−4^ S·cm^−1^ in hydrated samples according to González and Betzabe [27]. The power density reaches a maximum of 0.034 mW/cm^2^. This low value is a consequence of the fact that the compound is undoped.

Lue et al. [29] reported high power densities (15.3 mWcm^−2^) in a PVA/KOH system with fumed silica fillings in their study of a direct methanol alkaline fuel cell (DMAFCS). Additionally, Li et al. [30] reported a PVA/CS membrane with a power density of 67 mW/cm^−2^. Quaternized chitosan (Q-CS) and quaternized PVA (Q-PVA) were used in this study. The study suggests that the quaternary ammonium cations incorporated in the polymers function better than the primary amino groups present in the hydrated chitosan without modifications with regards to the mechanism of ionic conduction. García et al. [24] argue that in general, commercial alkaline anion exchange membranes stand out regarding good electrical behavior. Figure 9b shows the voltage–current curves for the Nafion^®^ 117 membrane included as a solid electrolyte in the fuel cell; here, the evaluation is carried out with the same humidity conditions established for the study compound. This proton exchange membrane has an energy efficiency in the range of 10% and 28.6% and an average voltage Vca = 836 mV. Because the membrane is a commercial proton exchange electrolyte, it presents a greater power density close to 5.5 mW/cm^2^ under the studied conditions.

## 5. Conclusions

The moisture absorption capacity of the PVA/CS- (xTiO_2_) compound can be controlled with the incorporation of the ceramic oxide. For concentrations greater than x = 1000 ppm, the hydrophobic properties of the membrane are promoted, while at low concentrations of nanofiller, the hydrophilic properties are favored, with moisture absorption of up to 90%. In the latter case, the hygroscopic TiO_2_ particles (average size 25 nm) act without competing with the water molecules by occupying the nanopores of the membrane. Thermal analysis by TGA indicates that a lower water loss (approximately 7% or less) is present in the PVA/CS compound (1000 ppm TiO_2_) with increases in temperature from 30 to 140 °C. This is due to the hygroscopic properties of the filler and its synergistic interaction with the ionic groups of the binary membrane. The PVA/CS compound (1000 ppm TiO_2_) displays a low activation energy (0.33 eV) at high temperature (90–160 °C). This is may be attributed to the plasticizing effect of the PVA and the adequate concentration of the dispersed filler in the CS matrix. The tensile tests of the membranes show that the compound has higher Young’s modulus and maximum resistance to rupture than the PVA. When the compound is doped (2 M KOH), both the toughness (50%) and percent elongation (36%) increase with respect to those of the undoped sample. This is due to the interaction of the KOH with the hydrogen bonds present in the binary membrane, producing a plasticizing effect in the compound. The PVA/CS compound (1000 ppm TiO_2_) is being validated as a solid polymeric electrolyte. The voltage–current tests performed in a proton exchange membrane fuel cell (PEMFC) reach an energy efficiency (17%) and open circuit voltage (1.0 V) comparable to those obtained for the commercial Nafion^®^ 117 membrane under similar conditions of moisture saturation (95% RH). Taking into account that the results are obtained for the undoped binary matrix, a low current density is observed. However, the compound can be considered promising for consideration in the development of a solid polymeric electrolyte. It is important to mention that, in a future work, it will be interesting to measure both the ionic conductivity of the electrolyte as a function of its humidity and the energy efficiency in a methanol fuel cell of the samples doped with KOH.
